# Rheology of vitrimers

**DOI:** 10.1038/s41467-022-33321-w

**Published:** 2022-09-30

**Authors:** Fanlong Meng, Mohand O. Saed, Eugene M. Terentjev

**Affiliations:** 1 CAS Key Laboratory for Theoretical Physics, Chinese Academy of Sciences, Beijing, 100190 China; 2 School of Physical Sciences, University of Chinese Academy of Sciences, Beijing, 100049 China; 3 Wenzhou Institute, University of Chinese Academy of Sciences, Wenzhou, Zhejiang 325001 China; 4 Cavendish Laboratory, University of Cambridge, JJ Thomson Avenue, Cambridge, CB3 0HE UK; 5 Cambridge Smart Plastics Ltd., 18 Hurrell Road, Cambridge, CB4 3RH UK

**Keywords:** Rheology, Chemical physics, Polymers

## Abstract

We describe the full rheology profile of vitrimers, from small deformation (linear) to large deformation (non-linear) viscoelastic behaviour, providing concise analytical expressions to assist the experimental data analysis, and also clarify the emerging insights and rheological concepts in the subject. We identify the elastic-plastic transition at a time scale comparable to the life-time of the exchangeable bonds in the vitrimer network, and propose a new method to deduce material parameters using the Master Curves. At large plastic creep, we describe the strain thinning when the material is subjected to a constant stress or force, and suggest another method to characterize the material parameters from the creep curves. We also investigate partial vitrimers including a permanent sub-network and an exchangeable sub-network where the bond exchange occurs. In creep, such materials can exhibit either strain thinning or strain thickening, depending on applied load, and present the phase diagram of this response.

## Abstract (web summary)

Rheology studies on vitrimers have mostly focused on their linear viscoelasticity under small deformations. Here, the authors develop a full rheological understanding of vitrimer response that spans between small deformation and large-deformation regime, and across 22 decades of effective frequency, providing clear and concise analytical expressions to assist the experimental data analysis and propose a method to deduce material parameters using Master Curves.

## Introduction

Transient polymer networks can exhibit excellent mechanical and thermal properties at low temperatures, and can also be reprocessable and recyclable at high temperatures as thermoplastics^1–3^. Traditionally, this concept referred to physically cross-linked networks, held by hydrogen bonds^4,5^, hydrophobic interactions^6,7^, or other self-assembled constraints^8–10^. However, in the last decade, there has been a rapid rise of examples of *dynamic covalent networks*, also called ‘covalent adaptable networks’, where the cross-links are exchangeable covalent bonds^11–13^. *Vitrimers*^14–16^ are a unique sub-class of the dynamic covalent networks, distinguished by the associative covalent bond exchange chemistry, where the total number of the covalent cross-links remains constant maintaining the material integrity. In other words, the bonds are only broken if new ones are already formed, as a single thermally activated process. As a result, the thermal viscosity of the materials changes with the temperature in the form of the Arrhenius law as in typical inorganic silica materials.

So far, the rheology studies on vitrimers have mostly focused on their linear viscoelasticity under small deformations^17–20^, where the responses of vitrimers resemble those of typical Maxwell materials: the storage modulus first increases with the deformation frequency and then saturates at a characteristic frequency *ω*_*s*_ (~1/*τ*, with *τ* as the life time of the cross-links as often probed by stress-relaxation tests^21–23^), and the loss modulus first increases and then decreases, peaked at the saturation frequency. Most of the studies in the literature use the method of stress relaxation to determine the characteristic relaxation time *τ*(*T*) and determine the activation energy of the thermal activated process using the Arrhenius plots and fitting. However, although theoretically rigorous, this method is vulnerable to several experimental and analysis errors related to the ambiguity of the time when the relaxation starts (*t* = 0), and how the relaxation time is determined. Also, polymer materials are often utilized in conditions of large deformations, which is especially important when there is an increasing plastic creep. Here one may wish to consider the ‘effective viscosity’ of a vitrimer in the plastic-flow regime, a notion that has attracted increasing attention in the analysis of recent experiments^21,24,25^, although the underlying mechanism of dissipation (and thus viscosity) is quite different from the classical fluids. It is important to develop a full rheological understanding of vitrimer response that spans between small-deformation elastic (or viscoelastic limit) and large-deformation (plastic flow) regime. We agree with the point recently made in the literature^26^ that, in such a rheologically complex material, multiple testing methods must be utilized, and made certain to agree with each other, for the full understanding of its response.

In this paper, we develop the fundamental theory of dynamic-mechanical response of vitrimers, and also of ‘partial vitrimers’ where a certain fraction of the network is cross-linked permanently, while the other fraction can exchange bonds by an associative reaction. The reason we have to do this is a number of emerging misconceptions in the field, the confusions caused by superficial analogies of, e.g., steady-state plastic deformation of solids and the viscous flow of liquids. There is also the problem of frequently mentioned ‘topology freezing temperature’ or ‘vitrification point’ *T*_*v*_, below which it is claimed that the bond exchanges are negligible. We find at least three definitions of *T*_*v*_ in the literature, ranging from the original Leibler’s criterion of where the effective viscosity crosses a value of 10^12^ Pa ⋅ s^14,15^ to the recent suggestion of Winter et al.^23^ identifying the point based on where the power-law relaxation of the modulus occurs, to the crossover point where the Arrhenius high-temperature relaxation regime changes to the glassy dynamics suggested by Guan et al.^27^. We cannot accept an empirical definition based on an arbitrary viscosity value, and we will make a point below to only deal with the ‘simple’ vitrimers where the single bond-exchange rate is the factor that controls the dynamics (hence no distribution of relaxation times and power-law dynamics). We also find a clear crossover in the Arrhenius plot of relaxation times, and thus find this definition of *T*_*v*_ the least objectionable. However, we show that the analysis of internal network dynamics based on the thermally activated exchange does not have any such transition temperature, and it is merely a matter of time (or deformation rate) at which the plastic flow is detectable—again in full analogy with silicate glass.

**Fig. 1:**
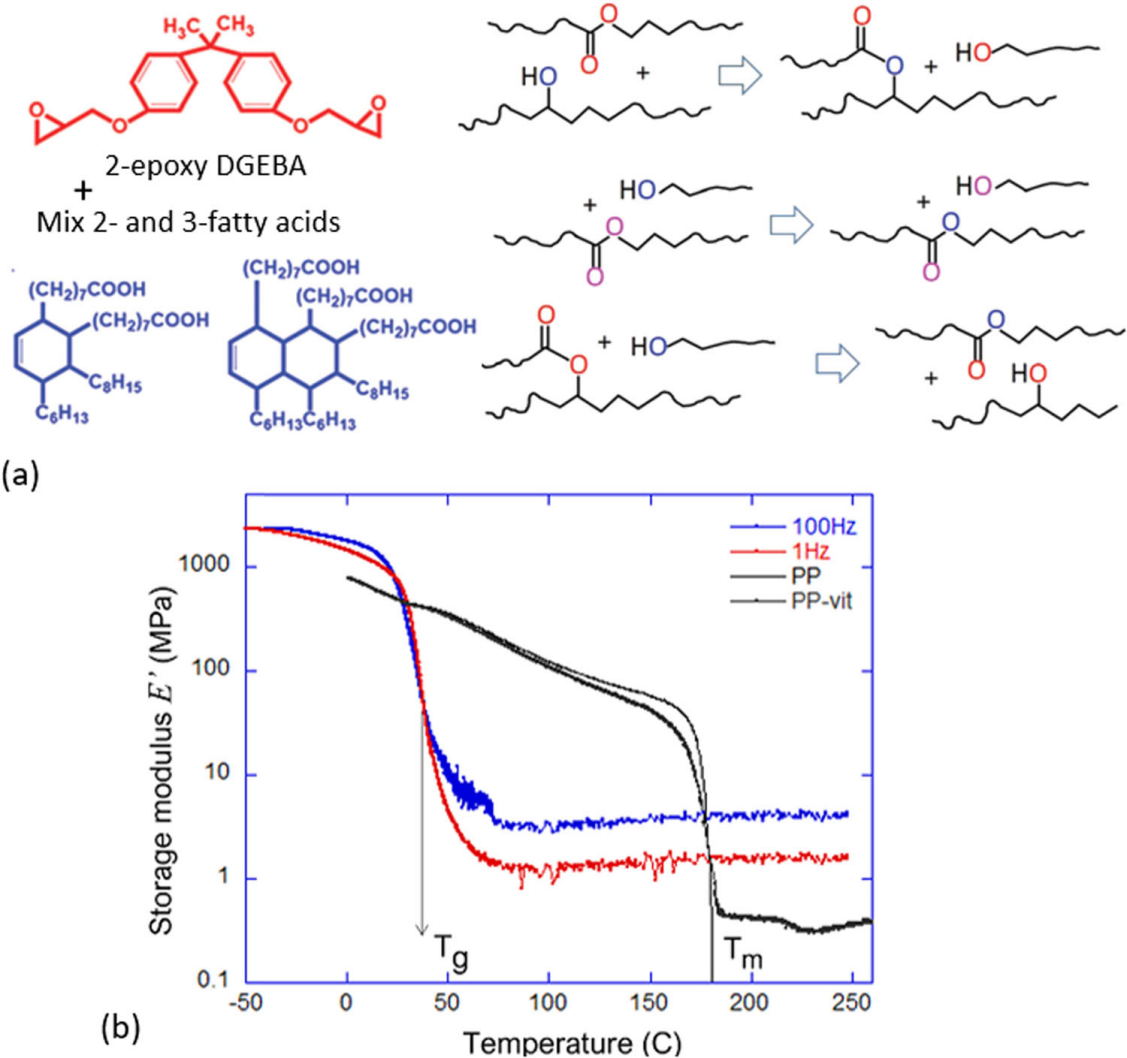
The benchamrk material and its key properties. **a** The components of the classical Leibler's vitrimer: the di-epoxy and a mix of 2- and 3-functional fatty acids, which bond into a cross-linked network at 130 °C with 5% of zinc catalyst (see refs. 14, 45 for details). Also shown is the scheme of transesterification bond exchange, reaching the equilibrium of 2-2 and 1-3 chain topology. **b** The typical linear dynamic response of a vitrimer. The first two curves show the storage modulus of the Leibler's benchmark epoxy vitrimer (at two frequencies of small oscillation), which shows the glass transition and the rubber modulus at high temperature. At these frequencies, the decrease in modulus is not yet showing. The other two curves are for comparison, showing the 5%-cross-linked polypropylene vitrimer and its precursor un-cross-linked polypropylene thermoplastic^46^, at 1 Hz. The low-temperature system is the same semicrystalline solid, regardless of dynamic cross-linking; the melt turns into a liquid above *T*_m_, while the vitrimer retains its rubber-elastic modulus.

In order to have a focused and controlled comparison with experimental data, we used the classical Leibler’s vitrimer concept^14^, based on the transesterification in the network formed by the epoxy-acid reaction, see Fig. 1a. This is the benchmark material, used in many subsequent studies, e.g., in 3D printing of vitrimers^28^, and we have deliberately prepared it for experimental testing of the theory predictions, as the most studied reference system. Our choice of the benchmark material is also dictated by the need to have a sufficiently slow bond-exchange rate. There are many examples in the literature when the vitrimers with quite readily exchangeable bonds were made, notably with borolate exchange^19,29–31^ as well as several other mechanisms, but when the exchange rate is comparable with internal rates of polymer chains relaxation in the network—the result is a complex distribution of relaxation times (resulting in power-law relaxation) that does not allow one to study the vitrimer dynamics cleanly. So we choose the Leibler’s network with relatively short strands to ensure the polymer relaxation times are shorter, and the catalyst conditions such that the transesterification is the only rate-limiting process that we want to study.

## Constitutive relation

In vitrimers, polymer chains can exchange at the cross-links or along the network strands with the rate of this process denoted as: $\beta={\omega }_{0}{e}^{-{W}_{0}/{k}_{{{{{{{{\rm{B}}}}}}}}}T}$^32,33^, where *ω*_0_ denotes the attempt rate of the exchange reaction (which is ~10^8^–10^9^ s^−1^ for ordinary small molecules^34^), and *W*_0_ denotes the energy barrier of bond exchange (which combines the association and dissociation steps). Note that the rate *β* is taken as independent of the material deformation here for seeking the transparency of the theory (see ref. 34 for details of deformation-dependent corrections). Moreover, the newly formed cross-links are assumed to appear in the relaxed state, i.e., they do not contribute to the elastic energy before applying any additional deformation. By considering the bond exchange reactions (see “Methods” for details), the total number of the cross-linked chains at time *t* is given by a combination of two terms: the continuously decreasing initial ones (*t* = 0, referring to those not experiencing any bond exchange), and the sum over the history of new cross-links (formed by bond exchange) at intermediate times $t^{\prime}\, < \, t$^34,35^:

1 $$N(t)={N}_{0}{e}^{-\beta t}+{N}_{0}\int\nolimits_{0}^{t}{{{{{{{\rm{d}}}}}}}}t^{\prime} \beta {e}^{-\beta (t-t^{\prime} )}\equiv {N}_{0},$$

which is a constant, one of key features of vitrimers. Correspondingly, the total elastic energy density of a vitrimer can be presented as a combination of two terms:

2 $$F(t)=F(t;0){e}^{-\beta t}+\int\nolimits_{0}^{t}{{{{{{{\rm{d}}}}}}}}t^{\prime} \beta {e}^{-\beta (t-t^{\prime} )}F(t;t^{\prime} ),$$

where $F(t;t^{\prime} )$ denotes the rubber-elastic energy density at the current time *t*, contributed by the chains cross-linked at time $t^{\prime}$. In this work, we use the simplest neo-Hookean model of rubber elasticity: $F(t;t^{\prime} )=\frac{1}{2}{G}_{0}({{{{{{{\rm{tr}}}}}}}}[{{{{{{{{\bf{E}}}}}}}}}^{{{{{{{{\rm{T}}}}}}}}}(t;t^{\prime} ){{{{{{{\bf{E}}}}}}}}(t;t^{\prime} )]-3)$, where *G*_0_ is the rubber (shear) modulus, and ${{{{{{{\bf{E}}}}}}}}(t;t^{\prime} )$ is the deformation gradient tensor at time *t* with respect to the reference state at time $t^{\prime}$, which can be related with the full deformation gradient tensor with respect to the reference state at *t* = 0 by the expression: ${{{{{{{\bf{E}}}}}}}}(t;t^{\prime} )={{{{{{{\bf{E}}}}}}}}(t;0){{{{{{{\bf{{E}}}}}}}^{-1}}}(t^{\prime} ;0)$. Apparently, the chains cross-linked at $t^{\prime}$ does not contribute to the elastic energy at $t^{\prime}$ with $F(t^{\prime} ;t^{\prime} )=0$, but the contribution becomes non-zero thereafter, $t \, > \, t^{\prime}$.

For the material undergoing a uniaxial stretching test, it elongates along ***e***_*x*_ with the stretching ratio *λ*(*t*), which can evolve with time, while the other two directions are free to contract incompressibly. In this geometry, the deformation gradient tensor is written as: ${{{{{{{\boldsymbol{E}}}}}}}}(t;0)=\lambda (t){{{{{{{{\boldsymbol{e}}}}}}}}}_{x}\otimes {{{{{{{{\boldsymbol{e}}}}}}}}}_{x}+1/\sqrt{\lambda }(t){{{{{{{{\boldsymbol{e}}}}}}}}}_{y}\otimes {{{{{{{{\boldsymbol{e}}}}}}}}}_{y}+1/\sqrt{\lambda }(t){{{{{{{{\boldsymbol{e}}}}}}}}}_{z}\otimes {{{{{{{{\boldsymbol{e}}}}}}}}}_{z}$. Suppose that the bonds exchange with the rate *β* and immediately re-cross-link into the force-free configuration. The constitutive relation relating the engineering tensile stress *σ*_*xx*_ and the stretching ratio *λ* of the deformed material can be obtained explicitly as:

3 $$\begin{array}{lll}{\sigma }_{xx}(t)&=&\frac{\partial F(t)}{\partial \lambda }={G}_{0}{e}^{-\beta t}\cdot \left[\lambda (t)-\frac{1}{{\lambda }^{2}(t)}\right]\\ &&+{G}_{0}\int\nolimits_{0}^{t}\,{{{{{{{\rm{d}}}}}}}}t^{\prime} \beta {e}^{-\beta (t-t^{\prime} )}\left[\frac{\lambda (t)}{{\lambda }^{2}(t^{\prime} )}-\frac{\lambda (t^{\prime} )}{{\lambda }^{2}(t)}\right],\end{array}$$

where *G*_0_ is the rubber (shear) modulus of a permanently cross-linked polymer network, for which *β* = 0. Other stress components are zero, if we assume free unconstrained surfaces: *σ*_*yy*_ = *σ*_*zz*_ = 0. This constitutive relation is too complicated to be of practical use in the analysis of experiment, which is why we now focus on compact analytical expressions emerging in key limiting cases.

## Results

### Linear oscillating viscoelasticity

By applying a small oscillatory deformation to the material, ${{{{{{{\boldsymbol{E}}}}}}}}(t;0) =(1+\epsilon \sin \omega t){{{{{{{{\boldsymbol{e}}}}}}}}}_{x}\otimes {{{{{{{{\boldsymbol{e}}}}}}}}}_{x}+(1-\epsilon \sin \omega t/2){{{{{{{{\boldsymbol{e}}}}}}}}}_{y}\otimes {{{{{{{{\boldsymbol{e}}}}}}}}}_{y}+(1-\epsilon \sin \omega t/2){{{{{{{{\boldsymbol{e}}}}}}}}}_{z} \otimes {{{{{{{{\boldsymbol{e}}}}}}}}}_{z}$, where *ϵ* = *λ* − 1 denotes an infinitesimal tensile strain, we can express the tensile stress [shown in Eq. (3)] in terms of the strain *ϵ* to its lowest order, i.e., *σ*_*xx*_ ~ *ϵ*, explicitly as (also see ref. 36):

4 $${\sigma }_{xx}(t) 	=3{G}_{0}\epsilon {e}^{-\beta t}\left[\sin \omega t+\,\int\nolimits_{0}^{t}\,{{{{{{{\rm{d}}}}}}}}t^{\prime} \beta {e}^{\beta t^{\prime} }(\sin \omega t-\sin \omega t^{\prime} )\right]\\ 	=3{G}_{0}\epsilon \left(\frac{{\omega }^{2}}{{\beta }^{2}+{\omega }^{2}}\sin \omega t\,+\,\frac{\beta \omega }{{\beta }^{2}+{\omega }^{2}}\cos \omega t\right)+{{{{{{{\rm{(d.\,t.)}}}}}}}}\\ 	=3\epsilon [G^{\prime} (\omega )\sin \omega t\,+\,G^{\prime\prime} (\omega )\cos \omega t]+{{{{{{{\rm{(d.\,t.)}}}}}}}},$$

where (d. t.) abbreviates for transient decaying terms proportional to *e*^−*βt*^, not surviving in the steady-state oscillatory response. The storage and the loss moduli in this deformation regime are:

5 $$G^{\prime} (\omega )={G}_{0}\frac{{\omega }^{2}}{{\beta }^{2}+{\omega }^{2}},\,\,G^{\prime\prime} (\omega )={G}_{0}\frac{\beta \omega }{{\beta }^{2}+{\omega }^{2}},$$

which is the classical result of the Maxwell viscoelasticity model, regarding 1/*β* as the relaxation time in the Maxwell model. Correspondingly, the loss factor of the material is: $\tan \delta=G^{\prime\prime} /G^{\prime}=\beta /\omega$. Obviously, the material responds elastically at short times, *t* ≪ 1/*β*, while the elastic-plastic transition happens at *t* ~ 1/*β*. Such linear viscoelasticity features have been experimentally tested on different vitrimers^18,20,26,37,38^. The rheological response described by the Maxwell model can well match how vitrimers respond in the low-frequency region, *ω* → 0, and the vitrimers experience glass transition at the peak of the loss factor in the high-frequency region. It is remarkable that after over 100 years of studies of Maxwell’s viscoelastic model, this is the first practical polymer system that genuinely and accurately follows this model. In retrospect, it is not surprising, given the single relaxation rate *β* that underpins all vitrimer kinetics. We must highlight again, that these conclusions are only valid when the bond-exchange rate *β* is sufficiently low, compared with other internal polymer relaxation rates, and thus remains the single rate-limiting process.

**Fig. 2:**
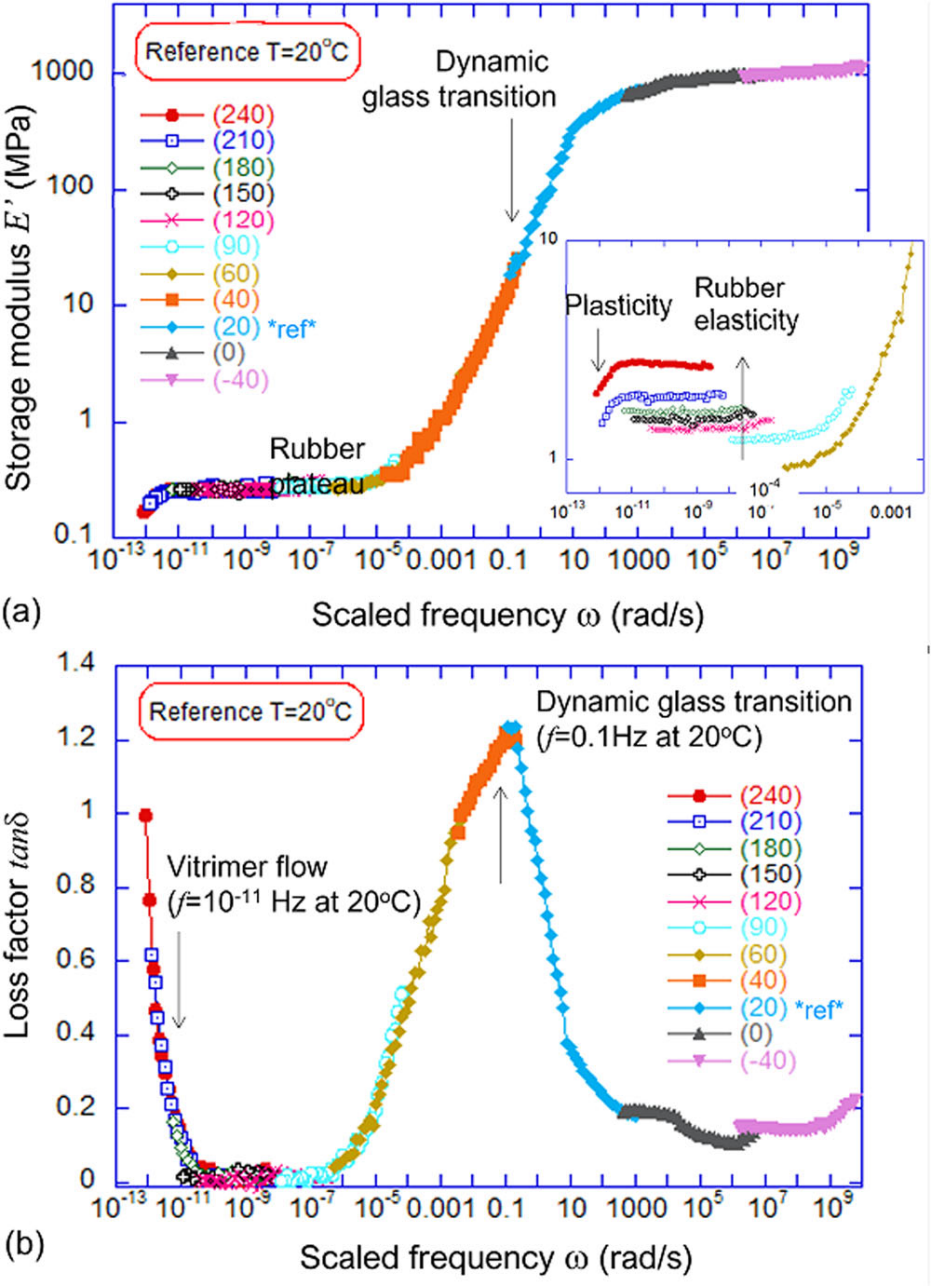
The Master Curves for the Leibler’s benchmark vitrimer, obtained by time-temperature superposition of frequency scans of the linear dynamic response at different temperatures. **a** The tensile storage modulus $E^{\prime}$ is presented as function of scaled frequency spanning the full range. Note that in the rubber-elastic regime the low-frequency rubber modulus grows with temperature, and needs to be scaled down proportionally for the proper Master Curve at 20 °C, see Inset. **b** The matching Master Curve for the loss factor $\tan \delta (\omega )$, for the reference *T* = 20 °C. Both plots show the dynamic glass transition, and the regime of vitrimer flow emerging at very low frequency.

Aiming to investigate rheological response of the vitrimers experimentally, we measured their linear dynamic-mechanical (oscillating) response, which leads to the familiar description in terms of a complex rubber modulus G*(*ω*), and used the time-temperature (*t*–*T*) superposition^39^ to construct Master Curves (see Fig. 2) both for the storage modulus (in this case the corresponding Young modulus $E^{\prime} (\omega )$ for tensile deformation) and the loss factor $\tan \delta (\omega )$. See the Supporting Information for more details on Master Curves construction and properties. Originally, the *t*–*T* superposition was developed to examine the glass transition of a material—and the vitrimer does show a prominent dynamic glass transition features (at an oscillation frequency of 0.1  Hz at 20 °C, or at 100 MHz when we re-scale the Master Curve for the reference 180 °C). But here we are even more interested in the other significant dynamical transformation that occurs in vitrimers: the elastic-plastic transition. Master Curves have never been constructed to combine the glass transition and the elastic-plastic transition, and so it is often confusing to the authors to deal with superposition in this high-temperature regime. This is technically challenging, as it requires collecting data at very high temperatures or very low frequencies, and it is theoretically challenging, as the response is affected by the classical effect of entropic rubber elasticity of the rubber modulus growing with temperature, see the inset of Fig. 2a. We encourage the interested reader to consult the Supporting Information, where more details of the *t*–*T* superposition of our data are listed. Specifically, having a clean set of data around the glass transition, our superposition does not require any vertical shifts in the modulus—although to superpose the modulus in the rubbery region we must indeed vertically shift (rescale) the modulus of different datasets to match the chosen reference temperature.

The loss factor $\tan \delta$ does not suffer from this difficulty of accounting for the temperature-dependent rubber modulus. It superposes naturally, and thus provides the guide for the shift factor (‘scaled frequency’ = *α*(*T*) ⋅ *ω*) to be used in the rubbery region for the modulus data. Nevertheless, this treatment is classical in polymer physics, and Fig. 2 clearly shows the onset of the elastic-plastic transition, on the same Master Curve that covers the glass transition as well. There are several rheological studies when this elastic-plastic transition was captured (mostly in the fast-exchange systems that does not allow simultaneous coverage of the glassy region).^23,26,40^ In our case, this transition happens at very low frequencies for the reference *T* = 20 °C. The reason that *t*–*T* superposition is able to capture this elastic-plastic transition is that, as with the glass transition, the intrinsic process rate is controlled by the single thermally activated process.

**Fig. 3:**
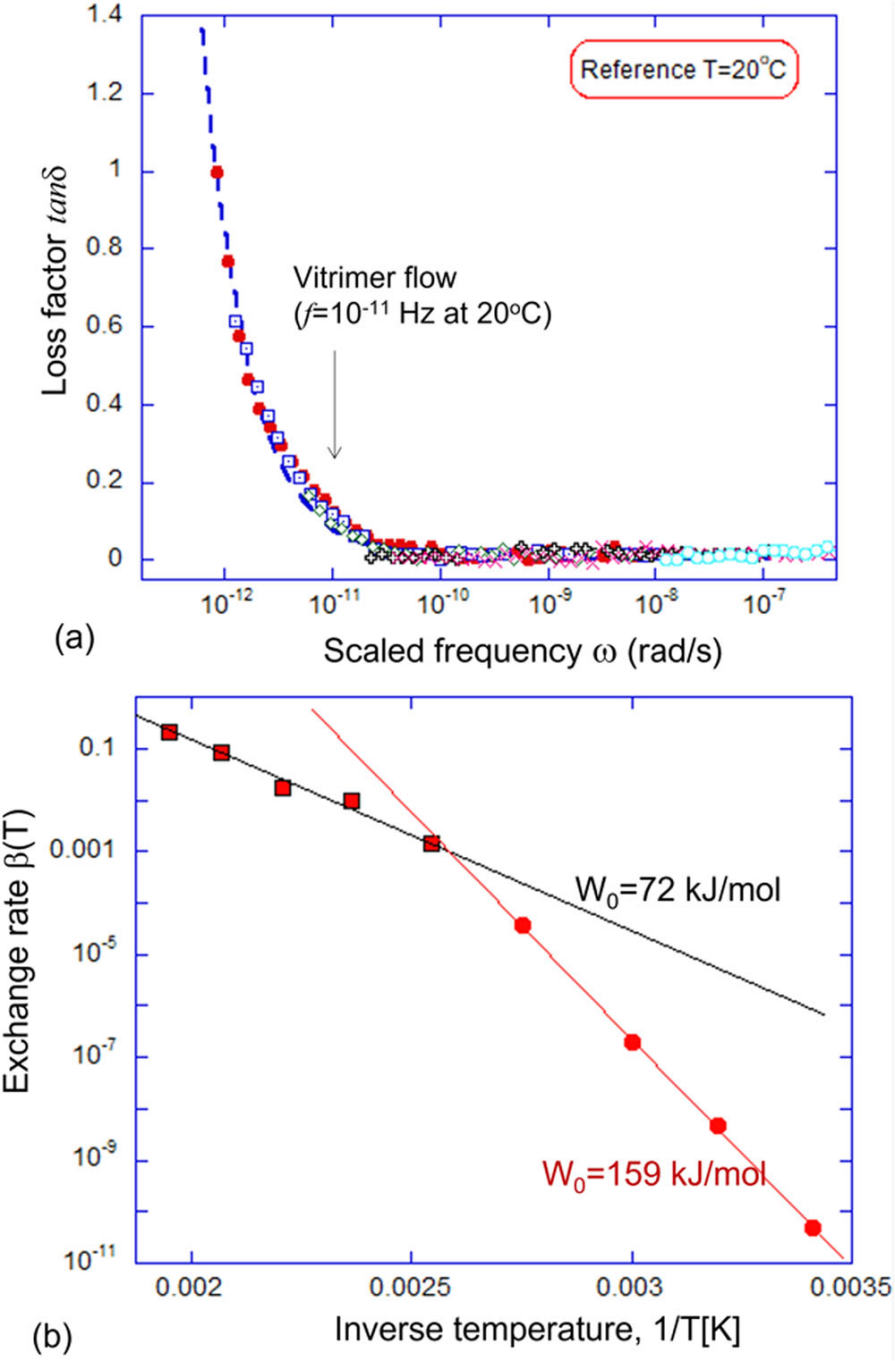
Analysis of elastic-plastic transition. **a** The fitting of the low-frequency `tail' of the Master Curve in Fig. 2b to the equation $\tan \delta (\omega )=\beta /\omega$ (blue dashed line). Carrying out this fitting for the same Master Curve re-scaled for various reference temperatures, we obtain a set of values for the model exchange rate *β*(*T*). **b** The Arrhenius thermal activation plot of *β*(*T*) against inverse temperature, the slope of which allows to find the activation energy. We found a dramatic change in this slope (and the activation energy), which gives *W*_0_ = 72 kJ/mol for the curves for high temperatures (*T* ≤ 120 °C)—but a much higher *W*_0_ = 159 kJ/mol for the lower temperature where the effects of the glass transition become noticeable.

The example is shown in Fig. 2b for *T*_ref_ = 20 °C, and its low-frequency region is enhanced in Fig. 3a, but we constructed such curves for many different reference temperatures. By fitting the low-frequency end of these $\tan \delta$ vs. *ω* curves with the simple equation, $\tan \delta=\beta /\omega$, Fig. 3a, we have obtained the exchange rates of our benchmark vitrimer, *β*(*T*). For instance, *β* = 0.1 s^−1^ at *T* = 210 °C, *β* = 0.016 s^−1^ at *T* = 180 °C, and *β* = 0.002 s^−1^ at *T* = 120 °C. One may question the virtue of such a fitting, which ignores the small but non-zero value of $\tan \delta$ in the rubber-elastic regime, but we found it very reliable since the rapidly rising 1/*ω* part is dominant in the data. Then, by assuming the Kramers thermally activated dynamics of the bond exchange reactions, i.e., $\beta={\omega }_{0}{e}^{-{W}_{0}/{k}_{{{{{{{{\rm{B}}}}}}}}}T}$, we can determine the energy barrier *W*_0_ for the bond exchange at high temperatures. The Arrhenius fitting gives us *W*_0_ = 1.2 × 10^−19 ^J = 72 kJ/mol. This value of *W*_0_ is very close to that reported in the original Leibler’s work^14^: *W*_0_ = 80 kJ/mol, where over 10 years ago it was measured from a completely different dataset of stress relaxation.

However, we also found that the scaled-frequency data for the reference temperatures below 90 °C give very different fitting values, see Fig. 3b, presumably because the raw data is affected by the material glass transition. So when we blindly re-scale the Master Curve frequencies to reference temperatures below 90 °C, the activation temperature dependence of the relaxation rate *β*(*T*) changes to a significantly higher activation energy of ca. 160 kJ/mol. We assume that additional (glassy) dynamics contributes here, while the pure transesterification exchange has the activation energy *W*_0_ given above. Note, that a similar crossover has been reported before, e.g., ref. 27, and we could similarly associate the crossover point in Fig. 3b with *T*_*v*_ = 122 °C in our case. But most of the authors tend to fit the low-temperature data with the empirical WLF dynamics^39^ or other more complex temperature-dependent forms^41,42^, while our point is that in many polymer-glass system (and certainly in our benchmark vitrimer) the data does not support the need for an additionl divergent WLF exponent with 1/(*T* − *T*_0_) and its extra fitting parameter: we have just four data points in Fig. 3b while many others have even less^18,27^, and we show here that the simple Arrhenius activation law is fitting the data perfectly. Discussing the glassy dynamics is not our aim here, but we need to caution from the often unnecessary use of WLF. We will now turn to the third independent experimental measure of bond exchange: the iso-stress plastic creep, which can only occur at elevated temperatures in reasonable time.

### Plastic creep in linear viscoelastic regime

The concept of effective viscosity is often used in the description of vitrimers in plastic flow regime, see refs. 14, 38. If we consider the material subjected to a constant tensile stress, which we denote *σ*_0_, the plastic creep will occur (at different rates depending on the temperature, see Fig. 2). The effective viscosity in the linear deformation regime can be defined in the following way: we write the tensile stress as

6 $${\sigma }_{0}=\int\nolimits_{0}^{t}dt^{\prime} \,3G(t-t^{\prime} )\frac{d\epsilon }{dt^{\prime} },$$

where the time-dependent (retarded) Young’s modulus is $E=3G(t-t^{\prime} )$ in this volume-conserving deformation geometry, well above the glass transition. The strain *ϵ*(*t*) in the time domain could be obtained from the Fourier-Laplace transformation of Eq. (6) to take the form:

7 $$\epsilon (t)={\sigma }_{0}\int\nolimits_{-i\delta -\infty }^{-i\delta+\infty }\frac{d\omega }{2\pi }\frac{{e}^{i\omega t}}{3\omega {G}{*}(\omega )}$$

where *δ* is an arbitrary positive constant defining the integration contour, and the complex shear modulus is defined as above, ${G}{*}(\omega )=G^{\prime} (\omega )+iG^{\prime\prime} (\omega )$. Now we expand it in Taylor series in powers of *ω*:

8 $${G}{*}(\omega )=i\omega \int\nolimits_{0}^{\infty }dt\,(1-i\omega t+...)G(t)=i\omega {g}_{0}+{\omega }^{2}{g}_{1}+...$$

with ${g}_{0}=\int\nolimits_{0}^{\infty }dt\,G(t)=\mathop{\lim }\nolimits_{\omega \to 0}G^{\prime\prime} (\omega )/\omega={G}_{0}/\beta$ and ${g}_{1}=\int\nolimits_{0}^{\infty }dt\,tG(t)$. Then the strain can be re-expressed as

9 $$\epsilon (t)={\sigma }_{0}\left(\frac{t}{3{g}_{0}}+\frac{{g}_{1}}{3{g}_{0}^{2}}\right),$$

and the steady-state effective viscosity of vitrimers has to be defined as:

10 $${\eta }_{0}=\frac{{\sigma }_{0}}{\dot{\epsilon }(t)}=3{g}_{0}=\frac{3{G}_{0}}{\beta }.$$

In fluids, the dissipation and viscous drag arise due to the pair correlation of interacting monomers, as they ‘push’ past each other in shear flow. In vitrimers, the plastic flow is controlled by the rate of bond exchange in the otherwise ‘solid’ elastic network. Both would show the characteristic thermal-activation behavior in the ‘viscosity’, but with the very different interpretation of activation energy. Considering the temperature dependence of the bond exchange rate, $\beta \propto {e}^{-{W}_{0}/{k}_{{{{{{{{\rm{B}}}}}}}}}T}$, we know the viscosity ${\eta }_{0}\propto {e}^{{W}_{0}/{k}_{{{{{{{{\rm{B}}}}}}}}}T}$, following the same Arrhenius law as the bond exchange. In contrast, the usual fluid viscosity, has the activation energy related to the pair interaction potential of molecules in the fluid^43^. These ideas were recently applied in the similar context in the important work on simulation of vitrimers^44^.

### Plastic creep to large deformation

**Fig. 4:**
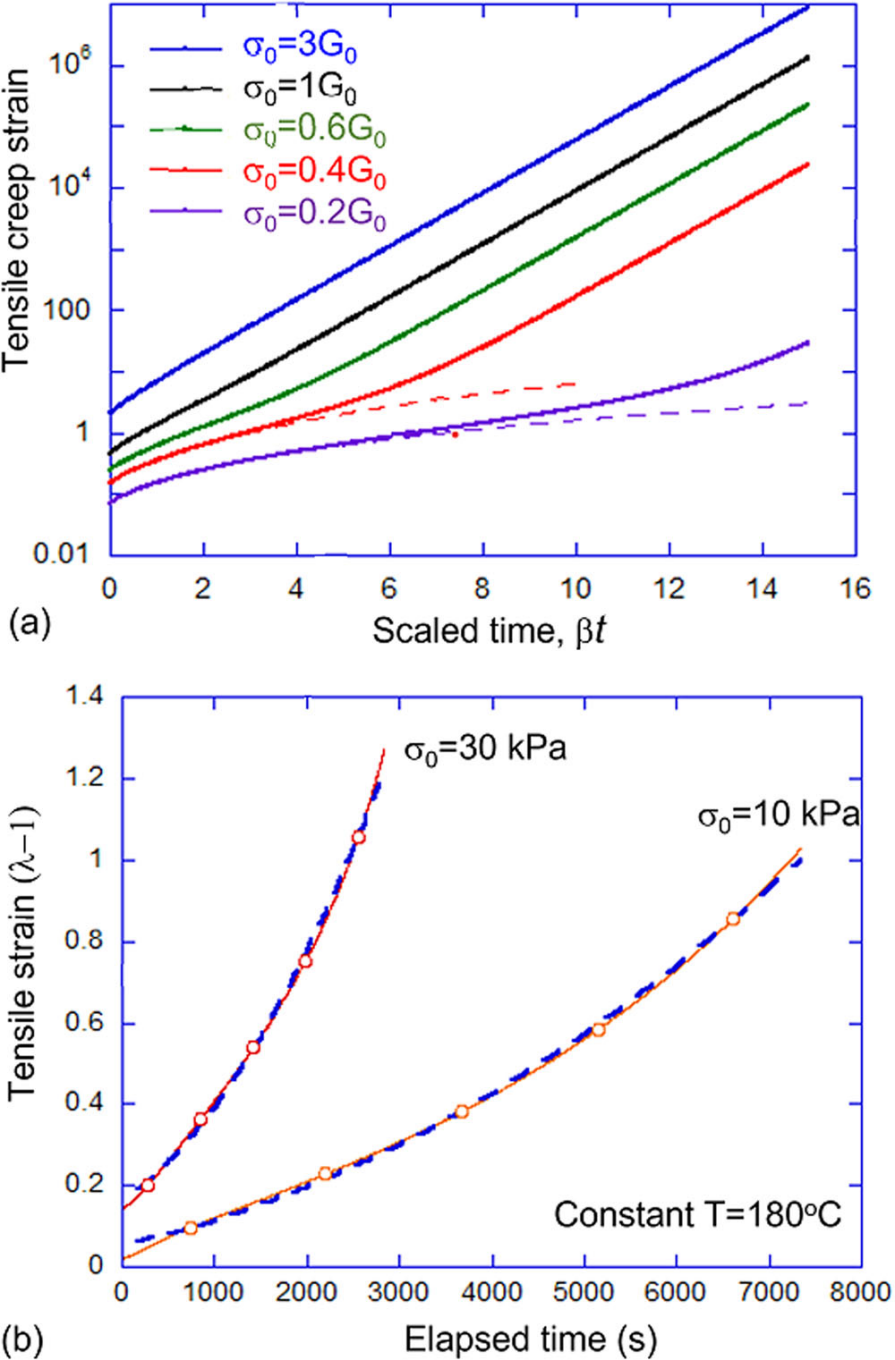
Strain evolution of vitrimers subjected to a constant engineering stress. **a** Comparison of the full numerical solution for *λ*(*t*) (solid lines) for different values of constant stress *σ*_0_. For comparison, the approximate expression (11) via the time-series (dashed lines) is shown for low-stress curves. **b** Experimental measurement of plastic creep under low constant stress, for the Leibler's benchmark vitrimer, at *T* = 180 ^∘^C. Dashed lines show the fitting with Eq. (11), giving the fitting parameter *β* = 0.016 s^−1^.

When subjected to a constant tensile stress, which we denote *σ*_0_, the vitrimer will creep continuously, eventually reaching large values of deformations. In this regime, the linearized treatment of the previous section will no longer be valid and the concepts of linear viscoelasticity do not apply. The full-time-dependent rheological response is encoded in Eq. (3), but we found no analytical way to invert it to obtain the full solution for strain *λ*(*t*) at constant stress *σ*_0_. This can be done numerically, and Fig. 4a shows example calculations of such plastic creep at constant tensile stress and temperature. One immediately notices the main feature, that at high external stress, the deformation moves as a simple exponential (highlighted by the log-scale of the plot). However, at low applied stress the initial non-exponential regime is evident.

Looking for analytical criteria of shear thinning, and estimates, we could carry out the approximate analysis assessing the deviation from the linear regime of Eq. (9). If the material is uniaxially stretched, the stretching ratio *λ*(*t*) in the short time limit (at *t* → 0) can be expressed in Taylor series:

11 $$\lambda (t;{\sigma }_{0})={\lambda }_{0}({\sigma }_{0})+{{{{{{{\mathcal{A}}}}}}}}({\sigma }_{0})t+{{{{{{{\mathcal{B}}}}}}}}({\sigma }_{0}){t}^{2}+{{{{{{{\rm{O}}}}}}}}({t}^{3}),$$

where *λ*_0_(*σ*_0_) is the instantaneous elastic deformation of the material upon the application of stress *σ*_0_ and can be obtained by solving ${\lambda }_{0}-1/{\lambda }_{0}^{2}={\sigma }_{0}/{G}_{0}$ for constant engineering stress (or simply *λ*_0_ = 1 + *σ*_0_/3*G*_0_ for small deformations). The first and the second expansion coefficients, ${{{{{{{\mathcal{A}}}}}}}}({\sigma }_{0})$ and ${{{{{{{\mathcal{B}}}}}}}}({\sigma }_{0})$, can be obtained by substituting the explicit form of *λ*(*t*) in Eq. (11) into the constitutive relation in Eq. (3), and implementing the constraint of engineering stress *σ*_*xx*_ = *σ*_0_ (or constant tensile force). We thus obtain

12 $${{{{{{{\mathcal{A}}}}}}}}=\beta \cdot \frac{{\lambda }_{0}({\lambda }_{0}^{3}-1)}{2+{\lambda }_{0}^{3}},$$

and

13 $${{{{{{{\mathcal{B}}}}}}}}={\beta }^{2}\cdot \frac{{\lambda }_{0}\left({\lambda }_{0}^{3}-1\right)\left({\lambda }_{0}^{6}-3{\lambda }_{0}^{4}+10{\lambda }_{0}^{3}-6{\lambda }_{0}-2\right)}{2{\left({\lambda }_{0}^{3}+2\right)}^{3}}.$$

If the deformation is small, then ${{{{{{{\mathcal{A}}}}}}}}\simeq \beta \cdot ({\lambda }_{0}-1)=\beta {\epsilon }_{0}$ and ${{{{{{{\mathcal{B}}}}}}}}\simeq {{{{{{{{\mathcal{A}}}}}}}}}^{2}$. Note that *λ*_0_(*σ*_0_), rather than the external parameter *σ*_0_ itself, is used in the expressions of ${{{{{{{\mathcal{A}}}}}}}}$ and ${{{{{{{\mathcal{B}}}}}}}}$ for simplicity. It is important that the second-order coefficient ${{{{{{{\mathcal{B}}}}}}}}$ is always positive, that is, ${{{{{{{\rm{d}}}}}}}}\eta /{{{{{{{\rm{d}}}}}}}}t\simeq -2{\sigma }_{0}{{{{{{{\mathcal{B}}}}}}}}({\sigma }_{0})/{{{{{{{{\mathcal{A}}}}}}}}}^{2}({\sigma }_{0}) \, < \, 0$ and the material is shear-thinning as is clear from both plots in Fig. 4.

This initial regime of plastic flow is plotted as dashed lines for the low-stress curves in Fig. 4a, and we see that eventually it transforms to the exponential growth of *λ*(*t*). The initial regime of Eq. (11) is barely detectable at *σ*_0_ > *G*_0_. We found a simple interpolation formula that accurately captures both regimes (see Supporting Information). The simplified expression takes the form:

14 $$\lambda (t)\,\approx\, {\lambda }_{0}({\sigma }_{0})+{{{{{{{\mathcal{A}}}}}}}}({\sigma }_{0})t+{{{{{{{\mathcal{B}}}}}}}}({\sigma }_{0}){t}^{2}+\frac{{\sigma }_{0}}{{G}_{0}}\left[{e}^{\beta t}-1\right].$$

Characteristically, the simple exponential with the rate of bond exchange *β* appears at every turn of the analysis of vitrimer rheology.

The shear-thinning ‘effective viscosity’ of the material is defined same as in Eq. (10), and takes the form: $\eta={\sigma }_{0}/{{{{{{{\mathcal{A}}}}}}}}({\sigma }_{0})\cdot [1-2{{{{{{{\mathcal{B}}}}}}}}({\sigma }_{0})/{{{{{{{\mathcal{A}}}}}}}}({\sigma }_{0})\cdot t]$. Figure 4b shows the comparison of the theoretical expression (Eq. (11)) with the creep experiments on our benchmark vitrimer. In these experiments, the initial strain step at *t* ≃ 0 was measured as *λ*_0_ = 1.005 for *σ*_0_ = 10 kPa, and *λ*_0_ = 1.014 for the stress of 30 kPa; this gives the rubber (shear) modulus of the material at that temperature: *G*_0_ ≃ 700 kPa. By fitting the experimental data with Eq. (11), we now have another way to estimate the bond exchange rate: *β* ≃ 0.016 s^−1^ at *T* = 180 °C, very close to what we had from fitting the Master Curve $\tan \delta (\omega )$ data earlier, which is very reassuring and confirms that the rheological theory developed here is self-consistent. The corresponding steady-state ‘effective viscosity’ of the material at 180 °C is: *η*_0_ = 3*G*_0_/*β* ≃ 1.3 × 10^8^ Pa ⋅ s. The same effective viscosity at 20 °C is predicted to be: ≃3 × 10^16^ Pa ⋅ s, clearly inaccessible to normal experiment. We considered ‘large deformation’ here, which may require to account for the finite extensibility of chains in the network, especially if the rate of bond exchange is low and the network chains stretch a lot before re-connecting. That is, we may need to replace neo-Hookean model of rubber elasticity that underpins Eq. (2) with a different model incorporating the finite stretchability, for instance the popular Gent model. However, we would not discuss this complication here to preserve the full transparency of the theory. 

### Partial vitrimers

Now we turn to another class of dynamic networks: partial vitrimers, which consist of two sub-networks—a typical vitrimer network allowing bond exchange and a permanent one without any bond exchange. The fraction of the vitrimer part is *ν* and that of the permanent elastic part is 1 − *ν*, respectively.

For the material undergoing a uniaxial stretching test, it elongates along ***e***_*x*_ with the stretching ratio *λ*(*t*) which can evolve with time, the derivation follows the steps outlined earlier, and produces the constitutive relation for the engineering tensile stress *σ*_*xx*_ and the stretching ratio *λ*:

15 $$\begin{array}{rcl}{\sigma }_{xx}(t;\nu )&=&G\,[(1-\nu )+\nu {e}^{-\beta t}]\cdot \left[\lambda (t)-\frac{1}{{\lambda }^{2}(t)}\right]\\ &&+\,G\,\nu \int\nolimits_{0}^{t}\,{{{{{{{\rm{d}}}}}}}}t^{\prime} \beta {e}^{-\beta (t-t^{\prime} )}\left[\frac{\lambda (t)}{{\lambda }^{2}(t^{\prime} )}-\frac{\lambda (t^{\prime} )}{{\lambda }^{2}(t)}\right].\end{array}$$

Other stress components are zero, if we assume free unconstrained surfaces in tensile test, i.e., *σ*_*yy*_ = *σ*_*zz*_ = 0. Note that the reference states for the cross-links in the permanent network part, and the cross-links in the exchangeable part of the network, which exist at the beginning *t* = 0 and have survived until the time *t*, are identical. This is reflected in defining the initial condition *λ*(*t* = 0) = 1, as shown in the first term of Eq. (15). However, the reference state for the cross-links which re-form at a certain time $t^{\prime}$ during the deformation in the vitrimer part is different, as was the case in pure vitrimers, and it is defined by $\lambda (t^{\prime} )$ in the second term of Eq. (15).

By applying a small oscillatory deformation to the material, and following the derivation steps that led to Eq. (4) the stress-strain relation now becomes:

16 $${\sigma }_{xx}=3\epsilon [G^{\prime} (\omega )\sin \omega t\,+\,G^{\prime\prime} (\omega )\cos \omega t]+{{{{{{{\rm{(d.\,t.)}}}}}}}},$$

where we ignore the transient decaying terms, and obtain the storage and the loss moduli of the partial vitrimer:

17 $$\begin{array}{r}G^{\prime} (\omega )=(1-\nu ){G}_{0}+\frac{\nu {G}_{0}\,{\omega }^{2}}{{\beta }^{2}+{\omega }^{2}},\,\,G^{\prime\prime} (\omega )=\frac{\nu {G}_{0}\,\beta \omega }{{\beta }^{2}+{\omega }^{2}}.\end{array}$$

Note the elastic plateau $G^{\prime} (\omega )=(1-\nu ){G}_{0}$ for partial vitrimers when *ω* → 0 (or *t* ≪ 1/*β*), and recall that there is no dissipation in the classical neo-Hookean rubber elasticity: the only loss arises from the bond exchange. Expressions (17) represent the classical Zener model of viscoelasticity.

Following the discussion on the creep response at constant stress *σ*_0_ in a full vitrimer, here we also present the analytical approximation for the evolving strain *λ*(*t*) in terms of time-series. In the partial vitrimer, such an expansion is only meaningful at the short times, the initial stages of plastic deformation, because at long times the strain will necessarily saturate at the constant value provided by the permanent network fraction with the final modulus (1 − *ν*)*G*_0_. However, the time series helps to assess the thinning vs. thickening response of the material at early stages of plastic creep:

18 $$\lambda (t;{\sigma }_{0},\ \nu )={\lambda }_{0}({\sigma }_{0})+{{{{{{{\mathcal{A}}}}}}}}({\sigma }_{0},\ \nu )t+{{{{{{{\mathcal{B}}}}}}}}({\sigma }_{0},\ \nu ){t}^{2}+{{{{{{{\rm{O}}}}}}}}({t}^{3}),$$

where, again, *λ*_0_ is the instantaneous stretching ratio of the material under a constant applied engineering stress *σ*_0_ (which is independent of vitrimer fraction *ν*): *λ*_0_ = 1 + *σ*_0_/3*G*_0_ for small deformations. Parameters ${{{{{{{\mathcal{A}}}}}}}}({\sigma }_{0},\, \nu )$ and ${{{{{{{\mathcal{B}}}}}}}}({\sigma }_{0},\, \nu )$ are the expansion coefficients, which now depend on the vitrimer fraction *ν*. We obtain

19 $${{{{{{{\mathcal{A}}}}}}}}({\sigma }_{0},\nu )=\beta \,\nu \,{\lambda }_{0}\frac{{\lambda }_{0}^{3}-1}{2+{\lambda }_{0}^{3}}$$

and

$${{{{{{{\mathcal{B}}}}}}}}({\sigma }_{0},\ \nu )={\beta }^{2}\nu \,{\lambda }_{0}\frac{(1-{\lambda }_{0}^{3})}{2{(2+{\lambda }_{0}^{3})}}+{\beta }^{2}{\nu }^{2}{\lambda }_{0}\frac{({\lambda }_{0}^{3}-1)(2{\lambda }_{0}^{6}-3{\lambda }_{0}^{4}+14{\lambda }_{0}^{3}-6{\lambda }_{0}+2)}{{2(2+{\lambda }_{0}^{3})}^{3}}.$$

Similarly, the ‘effective viscosity’ of the material is still defined as: $\eta={\sigma }_{0}/\dot{\lambda }$, and in the limit of *t* → 0, it is given by $\eta (t; {\sigma }_{0},\, \nu )\simeq {\sigma }_{0}/{{{{{{{\mathcal{A}}}}}}}}({\sigma }_{0},\, \nu )\cdot [1-2{{{{{{{\mathcal{B}}}}}}}}({\sigma }_{0},\, \nu )/{{{{{{{\mathcal{A}}}}}}}}({\sigma }_{0},\, \nu )\cdot t]$.

For a permanent elastic network, i.e., *ν* = 0, the result is simple: ${{{{{{{\mathcal{A}}}}}}}}({\sigma }_{0},\,\nu )={{{{{{{\mathcal{B}}}}}}}}({\sigma }_{0},\, \nu )=0$, meaning that for a permanent elastic network, there will be no further evolution of deformation after the instantaneous deformation. On the other hand, a full vitrimer with *ν* = 1 will exhibit strain thinning as discussed in the previous section.

**Fig. 5:**
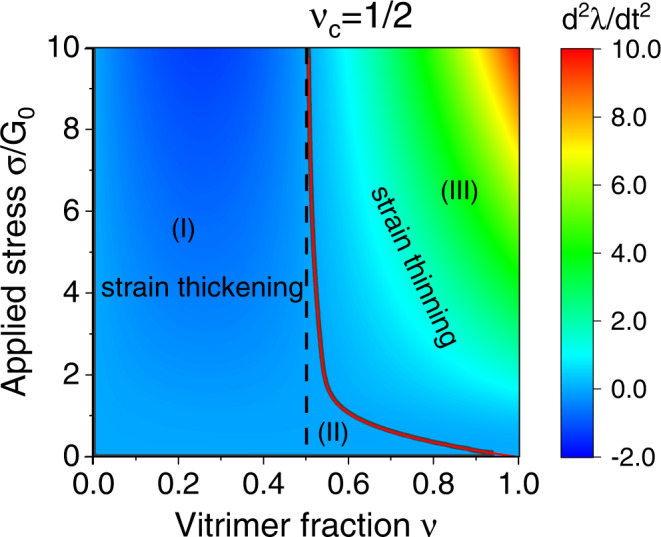
Acceleration, *dλ*^2^/*dt*^2^, of material extension in the short time limit, *t* → 0, in the space of (vitrimer fraction *ν*, stress). *ν*_*c*_ = 1/2 denotes the critical vitrimer fraction, below which the material can only exhibit strain thickening regardless of the applied stress. Positive and negative accelerations represent shear thinning [Region (III)] and thickening [Region (I) and (II)], respectively. The red line (the boundary between regions) is ν*(*σ*_0_) calculated from the full numerical solution, and the black line is represents the approximate low-deformation limit by Eq. (18); they overlap indicating the accuracy of approximation.

For materials with 0 < *ν* < 1, there exists a critical value *ν*_*c*_ = 1/2, below which the material can only exhibit strain thickening regardless of the magnitude of the tensile stress [Region I in Fig. 5]. This critical value can be obtained by assuming the material is stretched by a large tensile stress, when the second-order coefficient takes the limit ${{{{{{{\mathcal{B}}}}}}}}={\beta }^{2}\nu {\lambda }_{0}(\nu -1/2)$, thus the material exhibits strain hardening when *ν* < 1/2. For partial vitrimers with *ν* > *ν*_*c*_, the materials show strain thickening for small tensile stresses [Region II in Fig. 5] and strain thinning for large tensile stresses [Region III in Fig. 5]. The characteristic vitrimer fraction of strain thinning-thickening transition ν* for given stress *σ*_0_ is shown as the solid line in Fig. 5. In the limit of small stress, when the low-deformation limit [Eq. (18)] is valid, we find ν* = 1 − 2*σ*_0_/3*G*, and in the limit of large stress, i.e., *σ*_0_ ≫ *G*_0_, then ν* = *ν*_*c*_. Alternatively, one can obtain the characteristic stress of strain thinning-thickening transition, ${\sigma }_{0}^{*}$, from the solid line in Fig. 5 for the materials with given vitrimer fraction *ν*.

As discussed in previous sections, the full vitrimer with *ν* = 1 always exhibits strain thinning, and the acceleration of material extension increases with time under constant force (engineering stress), which is shown in Fig. 4. On the other hand, for purely elastic rubber with *ν* = 0, there is no further strain evolution after the instantaneous deformation (within the simple rubber-elastic model we employ here). If the vitrimer fraction of the materials is 0 < *ν* < *ν*_*c*_ = 1/2, the material can only show strain thickening, from *t* = 0 to *t* → *∞*.

**Fig. 6:**
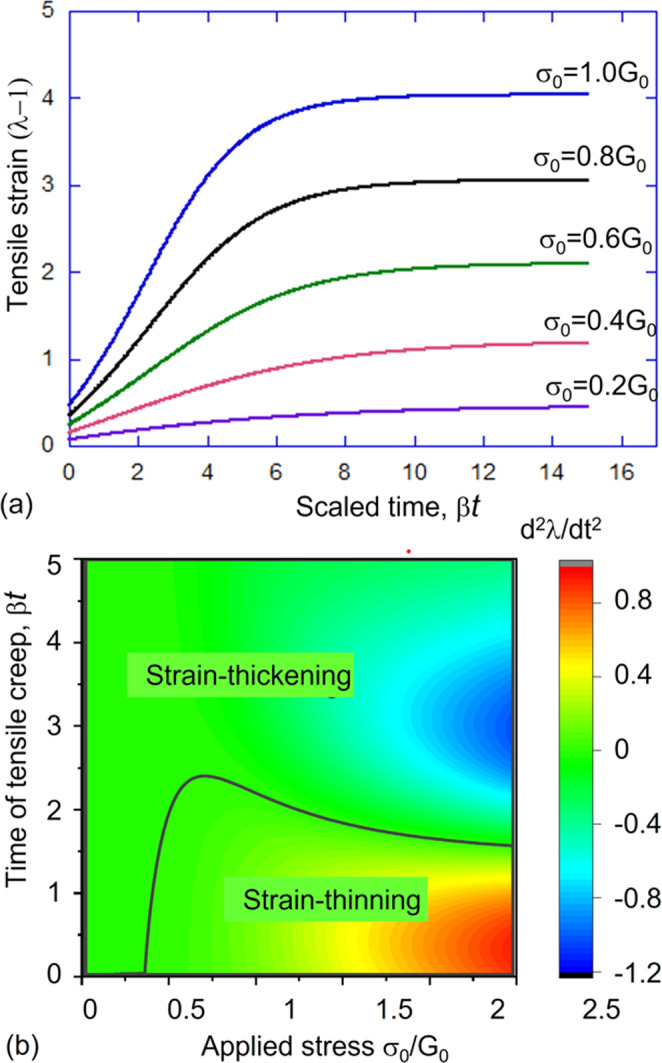
Rheological characteristics of partial vitrimers. **a** The strain evolution on plastic creep of partial vitrimer with the exchangeable fraction *ν* = 0.8, at different values of applied engineering stress (constant force), as labeled in the plot. At long times the deformation saturates at the level ${\lambda }_{\max }$ given in the text. **b** The `phase diagram' mapping the regions of strain thickening and strain thinning for different applied stress and time of creep, again for the partial vitrimer with *ν* = 0.8. Both plots are calculated numerically from the full solution of the constitutive Eq. (15).

The case for partial vitrimers of 1/2 < *ν* < 1 is more interesting, as shown in the ‘phase diagram’, Fig. 5. The material under constant load will exhibit strain thickening if the tensile stress is small, with the rate of plastic flow decelerating all the time until saturation at ${\lambda }_{\max }$. If the applied tensile stress is large, one finds a crossover from the thinning to thickening regime as the plastic creep progresses. This transition is easy to see in the changing sign of the second-order expansion coefficient ${{{{{{{\mathcal{B}}}}}}}}({\sigma }_{0},\nu )$ discussed above. In all cases, in the long time limit, *t* → *∞*, the tensile strain will reach the saturation value ${\lambda }_{\max }$, given by the solution of ${\lambda }_{\max }-1/{\lambda }_{\max }^{2}={\sigma }_{0}/[{G}_{0}(1-\nu )]$; in other words, the material behaves elastically in the long time limit. As always in vitrimers, the characteristic time for reaching saturation is determined by the bond-exchange rate *β*(*T*). All of these rheological features are summarized in Fig. 6.

## Discussion

We investigate the rheology of classical vitrimers, that is, polymer networks with associative bond exchange—and their ‘partial vitrimer’ counterparts where a fraction of the network is permanently elastic. Although the theory is complicated by retarded integral expressions accounting for the internal kinetics of network strands under load and relaxed, we aimed at presenting simple analytical expressions useful for practical application and analysis of experiment. Specifically, we examined two rheological regimes often found in experiment: the linear oscillating response, where we identified a close analogy with Maxwell materials, and the deformation (elastic and then creep) under constant load. In the latter case, we were able to offer analytical expressions for the creep strain at the early stages of iso-force deformation where many of the practical processing of vitrimers takes place. The results and fitting in Fig. 4b suggest that our approximate expressions are valid up to quite significant creep deformation. Examining the full large-deformation regime, we found that full vitrimers under high load deform with a simple exponential rate of flow, and offered an interpolation formula (14) for the crossover between the low-stress and high-stress regimes.

By applying this theory to experimental results obtained for the ‘benchmark vitrimer’, we took two methods of data analysis that give detailed information about material properties. In the small-strain oscillating regime, by constructing and analyzing Master Curves, we found a way to re-scale the low-frequency plastic-flow tail of the response (only visible at very high temperatures, due to the instrument limitations) to all temperatures, and thus find the rate of plastic flow (and the associated effective viscosity) at any temperature! Obviously, at lower temperatures, these are extremely low. For instance, the relaxation time for this benchmark epoxy-acid vitrimer (the inverse rate of plastic flow 1/*β*) is measured in minutes at *T* = 200 °C, but is around 600 years at *T* = 20 °C. However, we found an unexpected feature of this analysis: the mere shifting of Master Curve to different reference temperatures works well until the temperatures at which the glass transition effects become noticeable. The time-temperature superposition still works, as expected, but the activation energy emerging from the Arrhenius fitting of *β*(*T*) in the low-temperature region returns a very different energy barrier, which can be associated with the glassy dynamics as noted before^18,27,31^ (quite irrespective of the vitrimer rheology).

The second independent method to determine the intrinsic rate of bond exchange in the vitrimer is by the analysis of creep at constant load. Here, by fitting our theoretical expressions, we were able to determine the values of *β*, which matched the ones obtained in small-strain oscillating response—and by reflection, also the stress relaxation analysis in these materials. This reassures us about the validity of the theory, and the specific expressions designed to help with experimental analysis. The study of partial vitrimers was an extension of the main theory, where we also show the plastic-flow mediated transition from the fast elastic response of the full network to the slow elastic response of its permanent fraction alone. This crossover is controlled by the same intrinsic exchange rate *β* but changes its features depending on applied stress and the permanent network fraction. We hope that the use of exact, approximate, and interpolated equations given here, and the choice of several methods of experiment and analysis that could be used concurrently for validation, would help in practical development of vitrimer applications.

## Methods

### Sample preparation

To prepare the benchmark Leibler’s vitrimers, we strictly followed procedures in their 2011 work^14^. In brief, to obtain ‘soft vitrimer’, we mix the di-functional epoxy DGEBA with the combination of two- and three-functional fatty acids, Fig. 1a, calculating the equal molar ratio of epoxy and carboxylic acid reacting groups. The mixture (23% di-functional and 77% tri-functional) of carboxylic fatty acids (Pripol 1040) was kindly donated by CRODA. The 5mol% of zinc catalyst *Zn*(*Ac*)_2_ was added, and the regent mixed at 130 °C before pouring into a mould to react for at least 6 hours at 130°C. Supporting Information gives more details of the preparation.

### Linear viscoelasticity measurements

For dynamic-mechanical studies, we used the TA instrument DMA 850. Strips of the vitrimer, about 1-mm thick to avoid instrumental artefacts at too thick and too thin films, were mounted in tensile mode. For oscillating linear-response studies we run frequency scans at constant strain amplitude of 0.2% and fixed temperature, to obtain values $G^{\prime} (\omega )$ and $\tan \delta (\omega )$ for each temperature. These datasets were then used for time-temperature superposition explained in more detail in the Supplementary Information. For iso-force creep studies, the samples were equilibrated at a given temperature, after which a fast ramp of stress was applied to reach a set value *σ*_0_. After that, the change of strain with time was recorded while keeping the stress and temperature constant.

We deliberately did not employ stress-relaxation as characterization method, because it has been the method of choice by many authors, beginning from the original Leibler’s work. We have used it ourselves on other occasions, in full and partial vitrimers^35^, and are aware of several difficulties in data interpretation: the constant strain must be applied very fast, so no initial relaxation would take place during the ramp, and the effective *t* = 0 point is difficult to define (an error in this early time of relaxation affects the fitting very significantly).

### Internal kinetics

In vitrimers, polymer chains can exchange at the rate: $\beta={\omega }_{0}{e}^{-{W}_{0}/{k}_{{{{{{{{\rm{B}}}}}}}}}T}$^32,33^, where *ω*_0_ denotes the attempt rate of the exchange reaction (which is ~10^8^–10^9^ s^−1^ for ordinary small molecules^34^), and *W*_0_ denotes the energy barrier of bond exchange at the cross-links (or interpreted as that for bond association and dissociation process). Note that the rate *β* is taken as independent of the material deformation here for seeking the transparency of the theory (see ref. 34 for details of deformation-dependent corrections). Moreover, the newly formed cross-links are assumed to appear in the relaxed state, i.e., they do not contribute to the elastic energy before applying any additional deformation. Starting from the time *t* = 0, the total number of the cross-links is *N*_0_; after an infinitesimal time interval Δ*t*, the number of initial cross-links (referring to those not experiencing any bond exchange) will decrease to *N*_0_ ⋅ *e*^−*β*Δ*t*^, and meanwhile, the same number new cross-links will form with the cross-linked chains in the relaxed state (defined at time *t* = Δ*t*); the number of these new cross-links is *N*_0_ ⋅ *β*Δ*t* (a common characteristic of associative adaptive networks). If one repeats this bond exchange process at equal short intervals Δ*t*, then after *n* time intervals (with *t* = *n*Δ*t*), the number of initially cross-linked chains (those not experiencing any bond exchange) will decrease down to *N*_0_ ⋅ *e*^−*βn*Δ*t*^, and the number of chains cross-linked at some intermediate time *t* = *k*Δ*t* will decrease to *N*_0_ ⋅ *β*Δ*t* ⋅ *e*^−*β*(*n*−*k*)Δ*t*^. By summing up all cross-linked chains with different reference states defined at corresponding bond exchange time $t^{\prime}$ ($0\,\le \,t^{\prime} \,\le \,t$) and writing in a continuum form, the total number of the cross-linked chains at a time *t* is given by a combination of two terms: the continuously decreasing initial ones (those not experiencing any bond exchange), and the sum over the history of forming new cross-links at intermediate times $t^{\prime} \, < \, t$^34,35^:

20 $$N(t)={N}_{0}{e}^{-\beta t}+{N}_{0}\int\nolimits_{0}^{t}{{{{{{{\rm{d}}}}}}}}t^{\prime} \beta {e}^{-\beta (t-t^{\prime} )}\equiv {N}_{0}.$$

Correspondingly, the total elastic energy density of a vitrimer can be presented as a combination of two terms:

21 $$F(t)=F(t;0){e}^{-\beta t}+\int\nolimits_{0}^{t}{{{{{{{\rm{d}}}}}}}}t^{\prime} \beta {e}^{-\beta (t-t^{\prime} )}F(t;t^{\prime} ),$$

where $F(t;t^{\prime} )$ denotes the rubber-elastic energy density at the current time *t*, contributed by the chains cross-linked at time $t^{\prime}$.

## Supplementary information

Supplementary Information

## Data Availability

The data that support the findings of this study are available from the corresponding authors upon request.

## Code availability

The code that support the findings of this study are available from the corresponding authors upon request.

## References

[1] Yang Y, Urban MW (2013). Self-healing polymeric materials. Chem. Soc. Rev..

[2] Wei Z (2014). Self-healing gels based on constitutional dynamic chemistry and their potential applications. Chem. Soc. Rev..

[3] Wang S, Urban MW (2020). Self-healing polymers. Nature Rev. Mater..

[4] Stadler R, de Lucca Freitas L (1986). Thermoplastic elastomers by hydrogen bonding. Polymer Bull..

[5] Stadler, R. in *Permanent and Transient Networks* Vol. 75, 140–145 (Steinkopff, 1987).

[6] Rao Z, Inoue M, Matsuda M, Taguchi T (2011). Quick self-healing and thermo-reversible liposome gel. Coll. Surf. B: Biointerfaces.

[7] Tuncaboylu DC, Sari M, Oppermann W, Okay O (2011). Tough and self-healing hydrogels formed via hydrophobic interactions. Macromolecules.

[8] MacKnight WJ, Earnest TR (1981). The structure and properties of ionomers. J. Polym. Sci.: Macromol. Rev..

[9] Nakahata M, Takashima Y, Yamaguchi H, Harada A (2011). Redox-responsive self-healing materials formed from host-guest polymers. Nature Comm..

[10] Janeček ER (2015). Hybrid supramolecular and colloidal hydrogels that bridge multiple length scales. Angew. Chem. - Intl. Ed..

[11] Kloxin CJ, Bowman CN (2013). Covalent adaptable networks: smart, reconfigurable and responsive network systems. Chem. Soc. Rev..

[12] McBride MK (2019). Enabling applications of covalent adaptable networks. Annu. Rev. Chem. Biomolec. Eng..

[13] Bowman C, Du Prez F, Kalow J (2020). Introduction to chemistry for covalent adaptable networks. Polymer Chem..

[14] Montarnal D, Capelot M, Tournilhac F, Leibler L (2011). Silica-like malleable materials from permanent organic networks. Science.

[15] Capelot M, Montarnal D, Tournilhac F, Leibler L (2012). Metal-catalyzed transesterification for healing and assembling of thermosets. J. Am. Chem. Soc..

[16] Denissen W, Winne JM, Du Prez FE (2016). Vitrimers: permanent organic networks with glass-like fluidity. Chem. Sci..

[17] Zhou Y, Goossens JGP, Sijbesma RP, Heuts JPA (2017). Poly(butylene terephthalate)/glycerol-based vitrimers via solid-state polymerization. Macromolecules.

[18] Snijkers F, Pasquino R, Maffezzoli A (2017). Curing and viscoelasticity of vitrimers. Soft Matter.

[19] Ricarte RG, Cloître M, Tournilhac F (2020). Linear viscoelasticity and flow of self-assembled vitrimers: the case of a polyethylene/dioxaborolane system. Macromolecules.

[20] El-Zaatari BM, Ishibashi JSA, Kalow JA (2020). Cross-linker control of vitrimer flow. Polymer Chem..

[21] Guerre M, Taplan C, Nicolaÿ R, Winne JM, Du Prez FE (2018). Fluorinated vitrimer elastomers with a dual temperature response. J. Am. Chem Soc..

[22] Chen X, Li L, Wei T, Venerus DC, Torkelson JM (2019). Reprocessable polyhydroxyurethane network composites: effect of filler surface functionality on cross-link density recovery and stress relaxation. ACS Appl. Mater. Int..

[23] Fang H, Ye W, Ding Y, Winter HH (2020). Rheology of the critical transition state of an epoxy vitrimer. Macromolecules.

[24] Li L, Chen X, Jin K, Torkelson JM (2018). Vitrimers designed both to strongly suppress creep and to recover original cross-link density after reprocessing: quantitative theory and experiments. Macromolecules.

[25] Hubbard AM (2022). Creep mechanics of epoxy vitrimer materials. ACS Appl. Polymer Mater..

[26] Porath LE, Evans CM (2021). Importance of broad temperature windows and multiple rheological approaches for probing viscoelasticity and entropic elasticity in vitrimers. Macromolecules.

[27] Nishimura Y, Chung J, Muradyan H, Guan Z (2017). Silyl ether as a robust and thermally stable dynamic covalent motif for malleable polymer design. J. Am. Chem. Soc..

[28] Shi Q (2017). Recyclable 3d printing of vitrimer epoxy. Mater. Horiz..

[29] Röttger M (2017). High-performance vitrimers from commodity thermoplastics through dioxaborolane metathesis. Science.

[30] Saed MO, Gablier A, Terentejv EM (2019). Liquid crystalline vitrimers with full or partial boronic-ester bond exchange. Adv. Funct. Mater..

[31] Somanab B, Evans CM (2021). Effect of precise linker length, bond density, and broad temperature window on the rheological properties of ethylene vitrimers. Soft Matter.

[32] Tanaka F, Edwards SF (1992). Viscoelastic properties of physically cross-linked networks. transient network theory. Macromolecules.

[33] Tanaka F, Edwards SF (1992). Viscoelastic properties of physically crosslinked networks Part 2. Dynamic mechanical moduli. J. Non-Newt. Fluid Mech..

[34] Meng F, Pritchard RH, Terentjev EM (2016). Stress relaxation, dynamics, and plasticity of transient polymer networks. Macromolecules.

[35] Meng F, Saed MO, Terentjev EM (2019). Elasticity and relaxation in full and partial vitrimer networks. Macromolecules.

[36] Tanaka F, Edwards SF (1992). Viscoelastic properties of physically crosslinked networks. J. Non-Newt. Fluid Mech..

[37] Khedaioui D, Boisson C, D’Agosto F, Montarnal D (2019). Polyethylene aerogels with combined physical and chemical crosslinking: Improved mechanical resilience and shape-memory properties. Angew.Chem..

[38] Jourdain A (2020). Rheological properties of covalent adaptable networks with 1,2,3-triazolium cross-links: the missing link between vitrimers and dissociative networks. Macromolecules.

[39] Ferry, J. D. *Viscoelastic Properties of Polymers* 3rd edn (Wiley-VCH, 1980).

[40] Porath LE, Evans CM (2020). Cross-linker control of vitrimer flow. Polym. Chem..

[41] Elmatad YS, Chandler D, Garrahan JP (2009). Corresponding states of structural glass formers. J. Phys. Chem. B.

[42] Elmatad YS, Chandler D, Garrahan JP (2010). Corresponding states of structural glass formers. II. J. Phys. Chem. B.

[43] Kirkwood JG, Buff FP, Green MS (1949). The Statistical mechanical theory of transport processes. III. The coefficients of shear and bulk viscosity of liquids. J. Chem. Phys..

[44] Ricarte RG, Shanbhag S (2021). Unentangled vitrimer melts: interplay between chain relaxation and cross-link exchange controls linear rheology. Macromolecules.

[45] Capelot M, Unterlass MM, Tournilhac F, Leibler L (2012). Catalytic control of the vitrimer glass transition. ACS Macro Letters.

[46] Kar GP, Saed MO, Terentjev EM (2020). Scalable upcycling of thermoplastic polyolefins into vitrimers through transesterification. J. Mater. Chem. A.

