# Supplementary material for: Rheology of vitrimers
Source: Nat Commun. 2022 Sep 30;13:5753. doi: 10.1038/s41467-022-33321-w (PMC9525629; doi:10.1038/s41467-022-33321-w)
Supplement: Supplementary file 1 — Supplementary Information [file 41467_2022_33321_MOESM1_ESM.pdf]

Supplementary Information

Rheology of Vitrimers

Fanlong Meng, Mohand O. Saed and Eugene M. Terentjev

All the chemical products are commercially available and were used as received without further purification. Pripol 1040 was kindly provided by CRODA Smart Materials. It is a mixture of C18 carboxilic acids derivatives, containing about 23 wt% dimers and 77 wt% trimers. Di-functional epoxy DGEBA was purchased from Sigma Merck. Zinc acetate dihydrate, zinc acetylacetonate and glutaric anhydride were purchased from Acros Organics.

Soft vitrimer networks were synthesized from a mixture of dimers and trimers of fatty acids and DGEBA. In a first step, about 20g of the fatty acids (Pripol 1040, 296 g/molCOOH) and the catalyst (Zn(Ac)2, 2 H2O) at 5mol% to the carboxilic groups were introduced in a 100mL round-bottom flask. Temperature was increased to 130°C while maintaining the mixture under vacuum, until the catalyst particles were fully solubilized (2-3 h).

The fatty acid mixture containing solubilized catalyst, was mixed with DGEBA (174 g/mol of epoxy groups), with the stoichiometry between COOH and epoxy as 1:1, typically 15.75g of the mixture of fatty acids and catalyst and 9.25g of DGEBA. The mixture was kept at 130°C until full miscibility occurred, and then poured into a Teflon mold to keep for at least 6 h at 130°C for the epoxy-acid reaction to complete.

The frequency scans of dynamic-mechanical testing were carried out at various temperatures, maintaining the constant oscillation amplitude of 0.2%. The results for the storage tensile modulus E’(ω) and the loss factor tanδ(ω) are shown in Supplementary Figures 1 and 2.

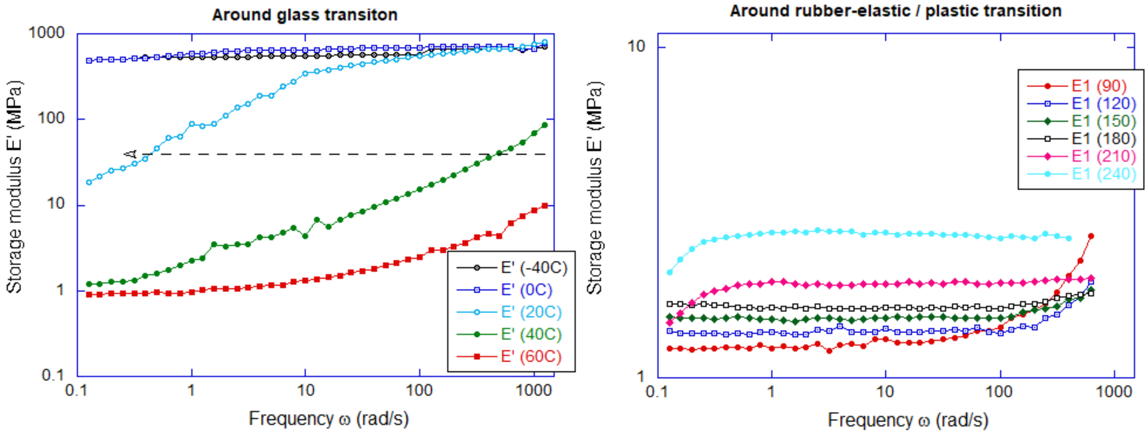

Supplementary Figure 1: The raw data of frequency scans at different temperatures showing the linear storage modulus E’(ω). Two plots are made to be able to see trends more clearly. In the low-temperature section of data one clearly sees the classical transition between the flat rubber-elastic plateau modulus of about 1 MPa, and the glass plateau modulus of near-GPa. In the high-temperature section, we can clearly see the increase of the rubber plateau modulus with temperature, which needs to be re-scaled back down to the plateau value expected at 20C. The two highest temperatures start showing the plastic flow in rapidly dropping values of storage modulus.

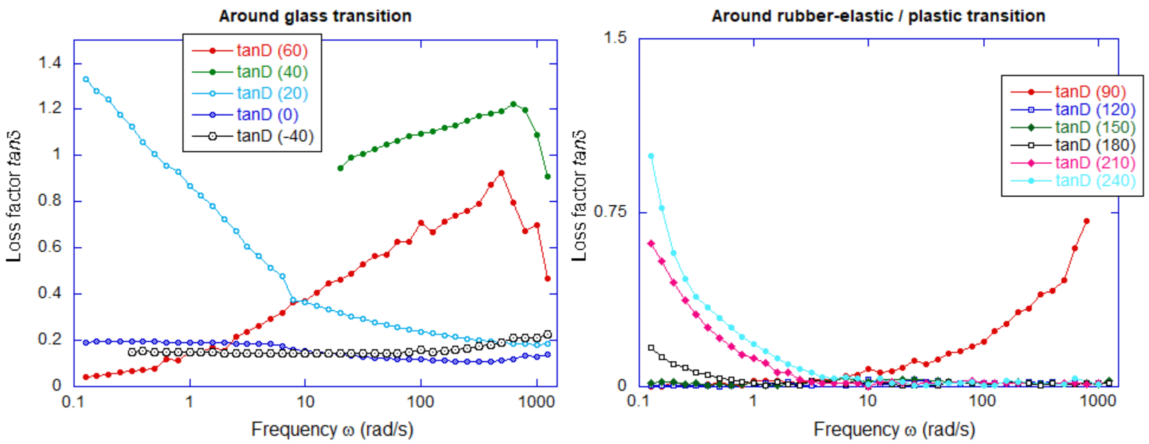

Supplementary Figure 2: The raw data of frequency scans at different temperatures showing the loss factor tan$\delta(\omega)$. As in Supplementary Figure 1, the two plots are made to see trends more clearly. In the low-temperature section of data one clearly sees the classical glass transition peak. In the high-temperature section, we start with the very low loss in the rubber-elastic state, but also the onset of plastic flow at the three highest temperatures. Unfortunately, the Master Curve constructed by time-temperature superposition of this data will not show the second peak at the characteristic frequency β, because that would require experiments at much higher temperatures where the sample integrity started to fail.

The Master Curves are obtained by shifting the raw data curves left and right along the frequency axis, which is achieved by multiplying the recorded frequency values by what is called the "shift factor" a(T), so the "scaled frequency" = a(T)·ω. In the classical WLF time-temperature superposition designed to cover the glass transition, the shift factor is expected to follow a certain temperature dependence (well publicized in the literature). In our case, the high-temperature data sets separately reflect the elastic-plastic transition of vitrimers, and we highlight those shift factors in Supplementary Figure 3:

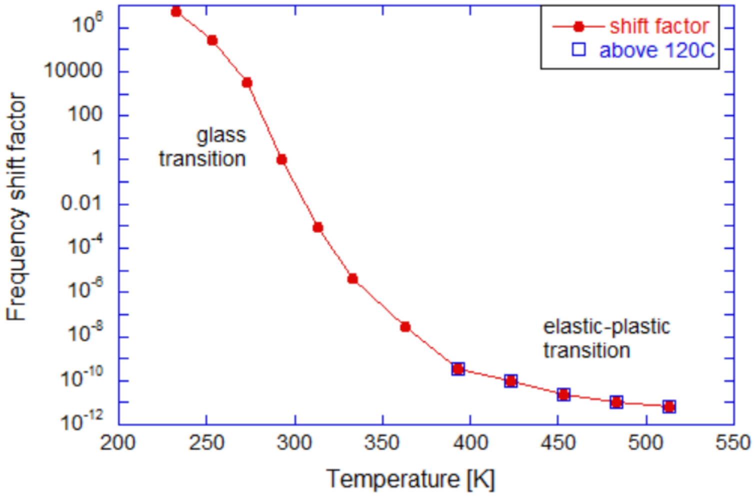

Supplementary Figure 3: The Log-Log plot of shift factors a(T) used to successfully superpose the raw frequency scans in both E'(ω) and the loss factor tan$\delta(\omega)$. The high-temperature region is highlighted, but the whole set of a(T) points represent a very clear trend. Unfortunately, we could not find a way to tie it to the WLF analysis or values, however, it does not diminish the value of the time-temperature superposition approach.

The interpolation formula for the creep tensile strain λ(t), presented in Eq.(14) of the main text relies on the following analysis. It needs to conform to the two limits of time: at short times, the Eq.(11) must be recovered:

$$\lambda(t) = \lambda_0 + A(\sigma_0)t + B(\sigma_0)t^2,$$

while at long times λ(t) must become a simple exponential growth with time:

$$\lambda(t) = \alpha \cdot \frac{\sigma_0}{G_0} e^{\beta t},$$

as evident from the plot in Supplementary Figure 4(a). However, we find that the prefactor α of this exponential time dependence strongly depends on the applied constant engineering stress σ_0. So we first found this prefactor numerically, for a range of values σ_0, and then attempted to model the relation $\alpha(\sigma_0)$: by fitting a curve to it:

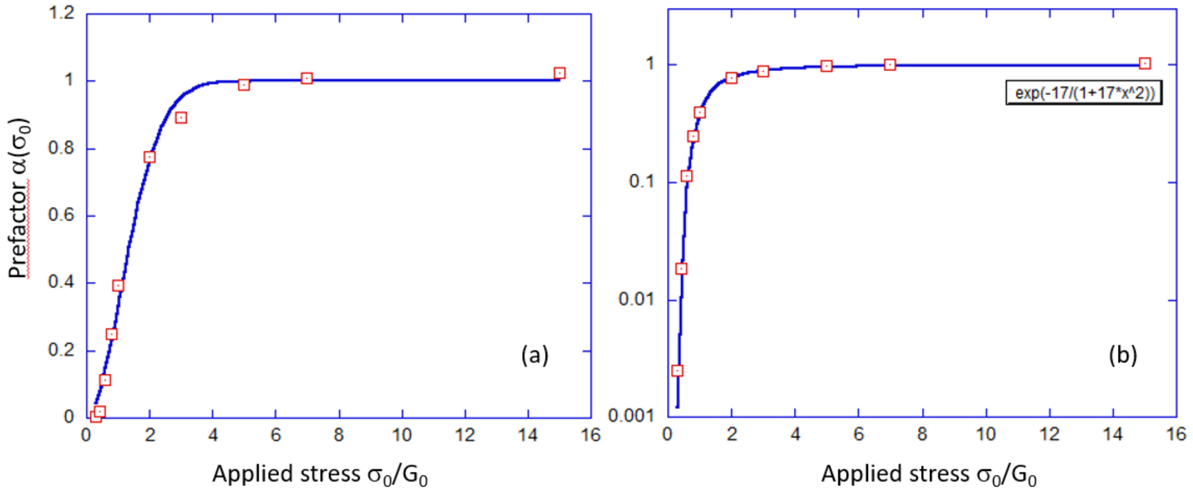

Supplementary Figure 4: Plotting the prefactor $\alpha(\sigma_0)$, calculated from the full numerical analysis, on the linear scale (a) and in log-linear scale (b). The sharp crossover that occurs at σ_0=G_0 is evident, and we achieve the best fit with the compressed exponential expression: $\alpha = \exp\left[-\frac{17}{1+17(\sigma_0/G_0)^2}\right]$.

The analysis in Supplementary Figure 4 tells that the exponential factor additive to the 'exact' small-strain expression for λ(t) in Eq.(11) vanishes rapidly both at short times (due to the (1-exp[-βt]) factor) and at small applied stress. Hence the accurate interpolation between these different limits is achieved by the rather cumbersome, although fully analytical expression:

$$\lambda(t) = \lambda_0 - \left(\frac{\sigma_0}{G_0}\right)\exp\left[-\frac{17}{1+17(\sigma_0/G_0)^2}\right] + \left[A(\sigma_0) - \beta\left(\frac{\sigma_0}{G_0}\right)\exp\left[-\frac{17}{1+17(\sigma_0/G_0)^2}\right]\right]t$$
$$+ \left[B(\sigma_0) - \frac{1}{2}\beta^2\left(\frac{\sigma_0}{G_0}\right)\exp\left[-\frac{17}{1+17(\sigma_0/G_0)^2}\right]\right]t^2$$
$$+ \left(\frac{\sigma_0}{G_0}\right)\exp\left[-\frac{17}{1+17(\sigma_0/G_0)^2}\right]e^{\beta t}$$

The truncated Eq.(14) given in the main text is just to highlight the ultimate limits of this full expression. The plots in Supplementary Figure 5 show how this analytical interpolation compares with

the exact numerical solution for the iso-stress creeping strain (analogous to what was shown in Supplementary Figure 4a in the main text):

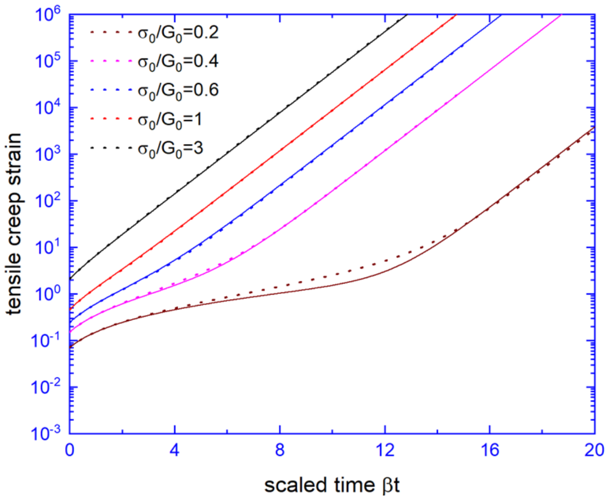

Supplementary Figure 5: Comparison of the exact numerical calculation of the iso-stress creep strain $\lambda(t)$-1, and the interpolated analytical expression derived from the fitted curve parameters (dotted lines).
